# A cuproptosis-related lncRNA signature for predicting prognosis and immunotherapy response of lung adenocarcinoma

**DOI:** 10.1186/s41065-023-00293-w

**Published:** 2023-07-24

**Authors:** Sheng Yu, Lingxue Tang, Qianqian Zhang, Wen Li, Senbang Yao, Yinlian Cai, Huaidong Cheng

**Affiliations:** 1 Department of Oncology, The Second Affiliated Hospital of Anhui Medical University, 678 Furong Road, Hefei, Anhui, 230601 China; 2 Shenzhen Clinical Medical School, Southern Medical University, Shenzhen, Guangdong China; 3 Department of Oncology, Shenzhen Hospital of Southern Medical University, Shenzhen, China

**Keywords:** Lung adenocarcinoma, Non-small cell lung cancer (NSCLC), Cuproptosis, LncRNAs, Prognostic signature, Immunotherapy response

## Abstract

**Background:**

Copper-induced cell death (cuproptosis) is a new regulatory cell death mechanism. Long noncoding RNAs (lncRNAs) are related to tumor immunity and metastasis. However, the correlation of cuproptosis-related lncRNAs with the immunotherapy response and prognosis of lung adenocarcinoma (LUAD) patients is not clear.

**Methods:**

We obtained the clinical characteristics and transcriptome data from TCGA-LUAD dataset (containing 539 LUAD and 59 paracancerous tissues). By utilizing LASSO-penalized Cox regression analysis, we identified a prognostic signature composed of cuproptosis-related lncRNAs. This signature was then utilized to segregate patients into two different risk categories based on their respective risk scores. The identification of differentially expressed genes (DEGs) between high- and low-risk groups was carried out using Gene Ontology (GO) and Kyoto Encyclopedia of Genes and Genomes (KEGG) enrichment analyses. We evaluated the immunotherapy response by analyzing tumor mutational burden (TMB), immunocyte infiltration and Tumor Immune Dysfunction and Exclusion (TIDE) web application. The "pRRophetic" R package was utilized to conduct further screening of potential therapeutic drugs for their sensitivity.

**Results:**

We ultimately identified a prognostic risk signature that includes six cuproptosis-related lncRNAs (AP003778.1, AC011611.2, CRNDE, AL162632.3, LY86-AS1, and AC090948.1). Compared with clinical characteristics, the signature was significantly correlated with prognosis following the control of confounding variables (HR = 2.287, 95% CI = 1.648–3.174, *p* ˂ 0.001), and correctly predicted 1-, 2-, and 3-year overall survival (OS) rates (AUC value = 0.725, 0.715, and 0.662, respectively) in LUAD patients. In terms of prognosis, patients categorized as low risk exhibited more positive results in comparison to those in the high-risk group. The enrichment analysis showed that the two groups had different immune signaling pathways. Immunotherapy may offer a more appropriate treatment option for high-risk patients due to their higher TMB and lower TIDE scores. The higher risk score may demonstrate increased sensitivity to bexarotene, cisplatin, epothilone B, and vinorelbine.

**Conclusions:**

Based on cuproptosis-related lncRNAs, we constructed and validated a novel risk signature that may be used to predict immunotherapy efficacy and prognosis in LUAD patients.

**Supplementary Information:**

The online version contains supplementary material available at 10.1186/s41065-023-00293-w.

## Highlights

1. Copper-induced cell death (cuproptosis) is a new mechanism of regulatory cell death that is different from apoptosis, necroptosis, pyroptosis, and ferroptosis.
2. Compared with clinical characteristics, a prognostic risk signature was identified that effectively predicted prognosis in LUAD patients.
3. Immunotherapy may be suitable for high-risk patients due to lower immune escape and higher TMB.

**Supplementary Information:**

The online version contains supplementary material available at 10.1186/s41065-023-00293-w.

## Introduction

Lung cancer seriously threatens people’s health, causing millions of global deaths per year [1]. Adenocarcinoma represents the highest proportion of lung cancer cases, totaling around 40% of histological types [2]. The chances of diagnosing LUAD at advanced stages are high, leading to an unfavorable prognosis with less than 20% of patients surviving beyond 5 years [3]. Survival prediction is essential for LUAD patients, and it determines the treatment strategy and living management. However, there are still limited accurate prognostic indicators, especially in LUAD patients.

Immunotherapeutic drugs represented by inhibitors of PD-1 and PD-L1 greatly prolong the survival duration of LUAD patients, but there is a lack of accurate predictive markers of response. Immunotherapy efficacy can be predicted through high expression levels of PD-L1 and TMB, but this effect may be limited in patients with low levels, despite some positive response to immunotherapy [4]. More accurate markers for predicting the immune response would enable the identification of more patients who may benefit from immunotherapy to benefit more patients. Cell death is necessary for the development and maintenance of homeostasis. The occurrence of apoptosis has been found to have a connection with immunotherapy resistance in cancer stem cells [5]. Inducing a combination of pyroptosis, ferroptosis, and necroptosis with immunotherapy has been shown to increase antitumor immunity [6].

According to recent research by Tsvetkov et al., cuproptosis induced by copper is a novel regulatory cell death mechanism [7]. All living organisms require copper, an essential trace element, similar to iron, and excessive concentrations produce cytotoxicity. Copper is involved in mitochondrial respiration, iron absorption, and antioxidant/detoxification processes [8]. Copper accumulation promotes tumor growth, angiopoiesis, and metastasis [9]. A decrease in serum copper levels has been related to the increase in interleukin (IL)-1β, IL6, IL4 and interferon-γ (IFN-γ) levels [10]. Research has indicated that copper promotes tumor PD-L1 expression and immune escape [11]. Copper accumulation promotes the inflammatory response via initiation of the NF-κB pathway [12]. Cuproptosis is associated with copper accumulation, but its role in cancer immunity remains unclear.

LncRNAs are RNA molecules longer than 200 nucleotides that do not code for proteins. LncRNAs carry out distinctive functions, including regulating transcription, controlling mRNA processing and modulating mRNA post-transcriptionally. LncRNAs are involved in mitochondrial function and glucose, glutamine and lipid metabolism and are closely related to cancer metabolism [13]. LncRNAs regulate tumor immunity and promote tumor metastasis [14]. Previous studies have shown that copper promotes lncRNA activation and autophagy [15]. Ferroptosis-related lncRNAs are involved in the cancer immune microenvironment [16]. Both ferroptosis and cuproptosis are metal-related forms of cell death. The impact of lncRNAs related to cuproptosis on tumor progression, especially in LUAD, are not clear, and more studies are urgently needed.

This research aimed to clarify the roles of cuproptosis-related lncRNAs in the immunotherapy response and prognosis of LUAD patients. We first randomly assigned TCGA-LUAD patients into two groups, consisting of a training group and a testing group. By utilizing cuproptosis-related lncRNA expression differences in the training group, we established a prognostic risk signature that was subsequently verified in both the testing group and entire patients. We additionally investigated the correlation between the risk signature and TMB, immune escape, and immunotherapy response, as well as its ability to predict sensitivity to potential antitumor drugs.

## Materials & methods

### Data collection

From the TCGA database (https://portal.gdc.cancer.gov/repository), we obtained the clinical characteristics and transcriptome data of LUAD patients (containing 539 LUAD and 59 paracancerous samples). Data for a total of 493 LUAD cases were enrolled after excluding patients with OS less than 30 days or missing data, including OS and survival status (alive or dead). The data cutoff was 2 May 2022. This study followed the public data access policies specified by the TCGA database and did not require ethical approval.

### Identification of cuproptosis-related lncRNAs

From Tsvetkov et al.'s work, we identified 19 genes associated with cuproptosis [7] (Supplementary Table 1). We performed a gene expression difference analysis using the "limma" R package, and evaluated the correlation between cuproptosis-related genes and their corresponding lncRNAs through Pearson correlation coefficients (cor > 0.4, *p* ˂ 0.001).

### Prognostic signature construction

We randomly assigned 493 cases into equal training and testing groups, and employed univariate Cox regression analysis in the training group to identify lncRNAs that were associated with prognosis. Furthermore, through the use of the "glmnet" R package and LASSO-penalized Cox regression analysis, we developed a prognostic signature that consisted of cuproptosis-related lncRNAs. Ultimately, based on the normalized expression levels of these lncRNAs and their respective regression coefficients, we determined a risk score for every patient. The following formula was used for the calculation:

$$\mathrm{Risk}\;\mathrm{Score}\:=\:0.3290\times\mathrm{AP}003778.1+0.5075\times\mathrm{AC}011611.2+(-0.2002)\times\mathrm{CRNDE}+1.8397\times\mathrm{AL}162632.3+(-1.9363)\times\mathrm{LY}86-\mathrm{AS}1+(-0.4078)\times\mathrm{AC}090948.1$$

Using the median risk score as a cutoff value, the training group was divided into two groups, and Kaplan–Meier (KM) analysis together with the log-rank test were used to compare OS between two groups. Using the "timeROC" R package, we evaluated the predictive capability of cuproptosis-related lncRNAs via time-dependent ROC curve analysis. Univariate and multivariate Cox regression analyses were utilized to investigate the connections between risk scores, clinical data, and prognosis. In the testing group and entire patients dataset, the prognostic model was further validated using the same cutoff value.

### Construction and evaluation of clinical nomogram

Based on clinical stage, T stage, gender, and risk score, a nomogram was created using the "RMS" R package, for the purpose of predicting the 1-, 2-, and 3-year OS. We employed the "ROC survival" R package to construct ROC curves in order to assess the efficacy of the nomogram, while decision analysis curves were generated using the "ggDCA" R package to evaluate its clinical usefulness, and calibration curves were created using the "rms" R package to gauge its precision.

### Functional enrichment analysis

GO and KEGG enrichment analyses were used to analyze the DEGs in the two risk groups by using the "clusterPofolier" R package. GO analysis revealed the participation of cuproptosis-related lncRNAs in biological processes (BP), molecular functions (MF), and cellular components (CC), while KEGG analysis identified the signaling pathways. Statistical significance was achieved with a false discovery rate < 0.05 and *p* < 0.05.

### Immune infiltration and immunotherapy response

The immune infiltration of two risk groups was compared by using the “GSVA” and “GSEABase” R packages. TIDE is an online public web application for analyzing immune escape and evaluates patient response by utilizing multiple transcriptomic biomarkers based on pretreatment tumor expression profiles (http://tide.dfci.harvard.edu/) [17]. We used TIDE to predict the possible response to immune checkpoint inhibitors (ICIs). Patients with high scores showed poor ICI treatment response. TMB is the quantity of mutations (including coding errors, base substitutions, insertions or deletions) per megabase (Mut/Mb) of DNA.

### Screening potential drugs

Gene expression is related to drug sensitivity, especially the 50% inhibitory concentration (IC_50_). The "pRRophetic" R package was utilized in our study to explore the IC_50_ of various antitumor drugs by employing risk scores, and has been demonstrated as effective in numerous clinical trials [18].

### Statistical analysis

Statistics were analyzed by R version 4.2.0. The correlation between the risk score and specific lncRNA expression was evaluated using Pearson's correlation analysis. Survival analysis was conducted by using KM curves and log-rank tests. The prognostic independence was assessed through univariate and multivariate Cox regression. The reliability and sensitivity of the signature were evaluated by using ROC curve analysis. All *p* values were considered two-tailed, and statistical significance was defined as *p* < 0.05.

## Results

### The clinical characteristics of patients in the training group were consistent with those in the testing group

Data for a total of 493 LUAD cases were enrolled and randomly assigned into a training group and a testing group. The clinical characteristics illustrated in Table 1 were no notable differences between the two groups.

**Table 1:** Clinical characteristics of LUAD patients in the testing and training groups

Covariates	Type	Total *n*(%)	Group *n*(%)		*P*-value
			Testing	Training	
Age (years)	< = 65	232(47.06%)	120(48.78%)	112(45.34%)	0.5527
	> 65	251(50.91%)	122(49.59%)	129(52.23%)	
	unknow	10(2.03%)	4(1.63%)	6(2.43%)	
Gender	female	264(53.55%)	130(52.85%)	134(54.25%)	0.8239
	male	229(46.45%)	116(47.15%)	113(45.75%)	
Tumor Stage	I	265(53.75%)	134(54.47%)	131(53.04%)	0.8863
	II	116(23.53%)	60(24.39%)	56(22.67%)	
	III	79(16.02%)	38(15.45%)	41(16.6%)	
	IV	25(5.07%)	11(4.47%)	14(5.67%)	
	unknow	8(1.62%)	3(1.22%)	5(2.02%)	
Stage					
T	T1	164(33.27%)	71(28.86%)	93(37.65%)	0.1079
	T2	265(53.75%)	143(58.13%)	122(49.39%)	
	T3	43(8.72%)	19(7.72%)	24(9.72%)	
	T4	18(3.65%)	11(4.47%)	7(2.83%)	
	unknow	3(0.61%)	2(0.81%)	1(0.4%)	
M	M0	327(66.33%)	159(64.63%)	168(68.02%)	0.9582
	M1	24(4.87%)	11(4.47%)	13(5.26%)	
	unknow	142(28.8%)	76(30.89%)	66(26.72%)	
N	N0	319(64.71%)	161(65.45%)	158(63.97%)	0.3212
	N1	93(18.86%)	50(20.33%)	43(17.41%)	
	N2	68(13.79%)	30(12.2%)	38(15.38%)	
	N3	2(0.41%)	0(0%)	2(0.81%)	
	unknow	11(2.23%)	5(2.03%)	6(2.43%)	

### Identification of prognostic signature in the training group

**Fig. 1:**
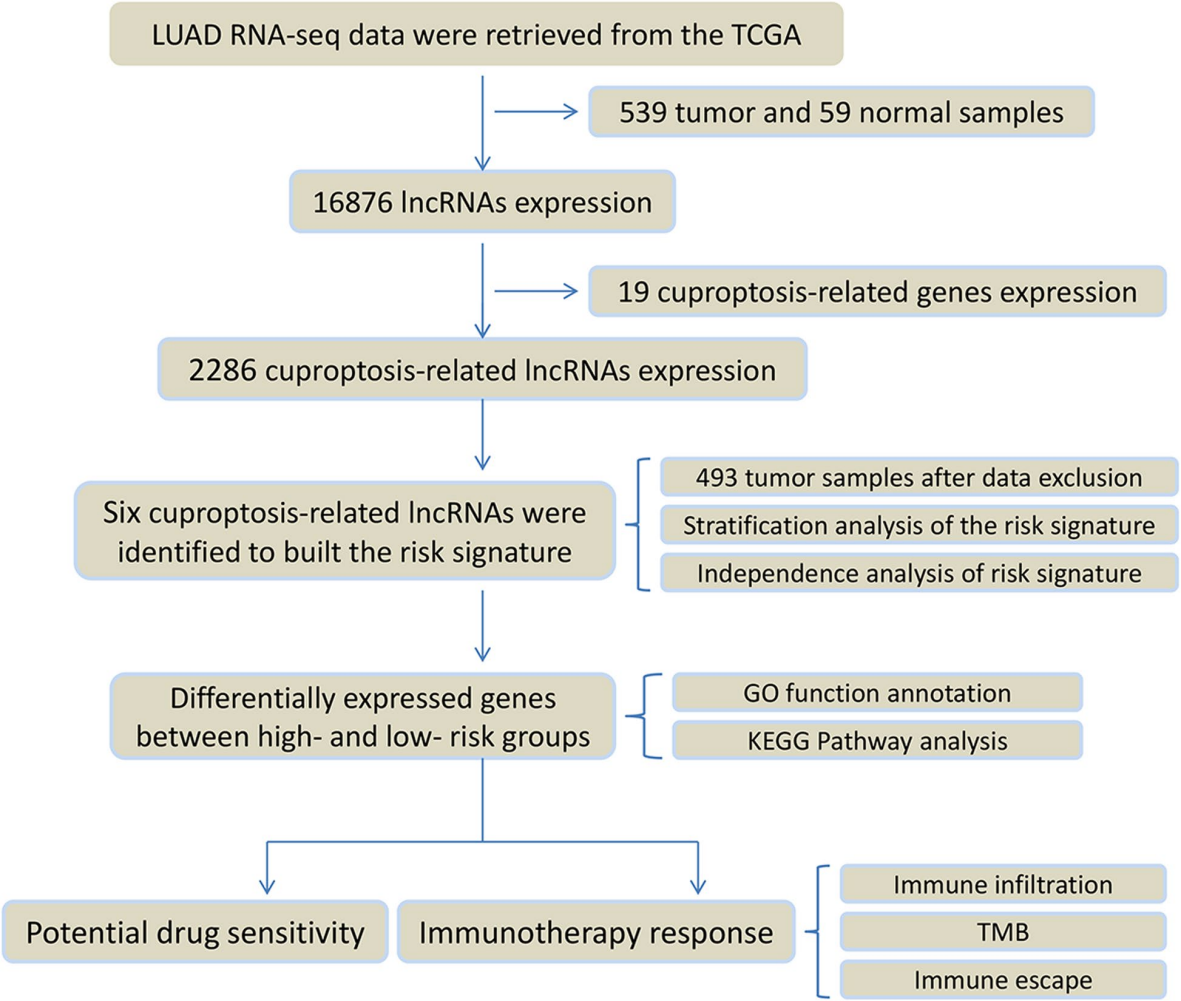
The flowchart of this study

**Fig. 2:**
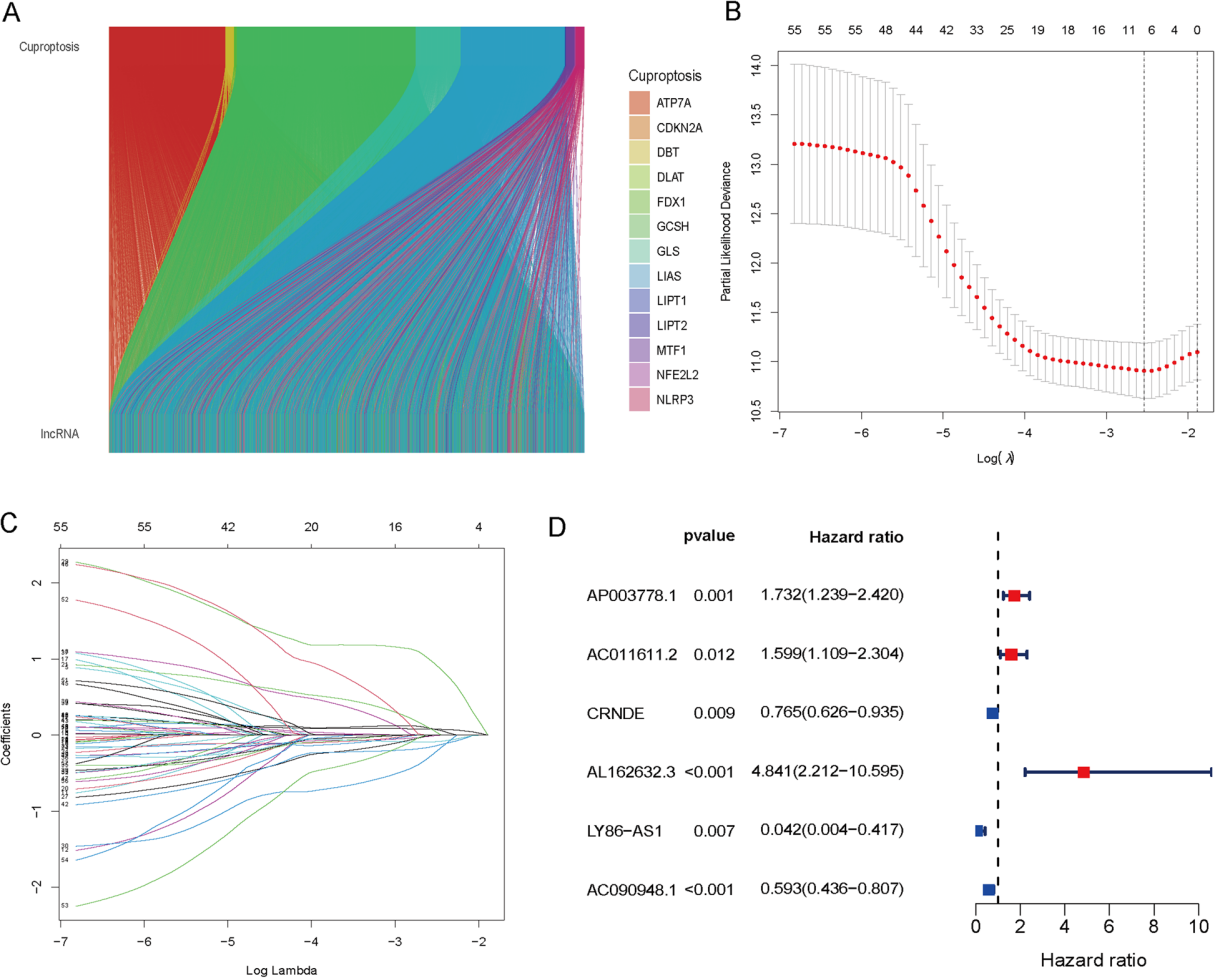
A prognostic signature containing six cuproptosis-related lncRNAs was constructed for LUAD patients. **A** The Sankey diagram shows the correlation between the expression of 13 cuproptosis-related genes and various lncRNAs in LUAD patients. **B**, **C** LASSO coefficient values and vertical dashed lines were calculated at the best log (lambda) value. **D** The forest map shows the relationship between 38 cuproptosis-related lncRNAs and prognosis by using univariate Cox regression analysis. Red indicates a positive correlation, while green indicates the opposite

The study's process is illustrated in Fig. 1. We obtained 19,938 mRNA expression data points and 16,876 lncRNA expression data points by collating the transcriptome sequencing data. A total of 2286 lncRNAs were identified as being cuproptosis-related based on expression correlation with 19 cuproptosis-related genes (cor > 0.4, *p* ˂ 0.001). As shown in Fig. 2A, the Sankey diagram demonstrates the close association of 13 cuproptosis-related genes with various lncRNAs. The 2286 cuproptosis-related lncRNAs were subjected to univariate Cox regression analysis to screen 57 lncRNAs associated with prognosis (*p* ˂ 0.05) (Fig. 2B, C), and six lncRNAs (AP003778.1, AC011611.2, CRNDE, AL162632.3, LY86-AS1, and AC090948.1) were ultimately identified as the prognostic risk signature by using LASSO analysis and cross validation (Fig. 2D, Supplementary Fig. 1).

### Prognostic signature validation in the testing group and entire patients

**Fig. 3:**
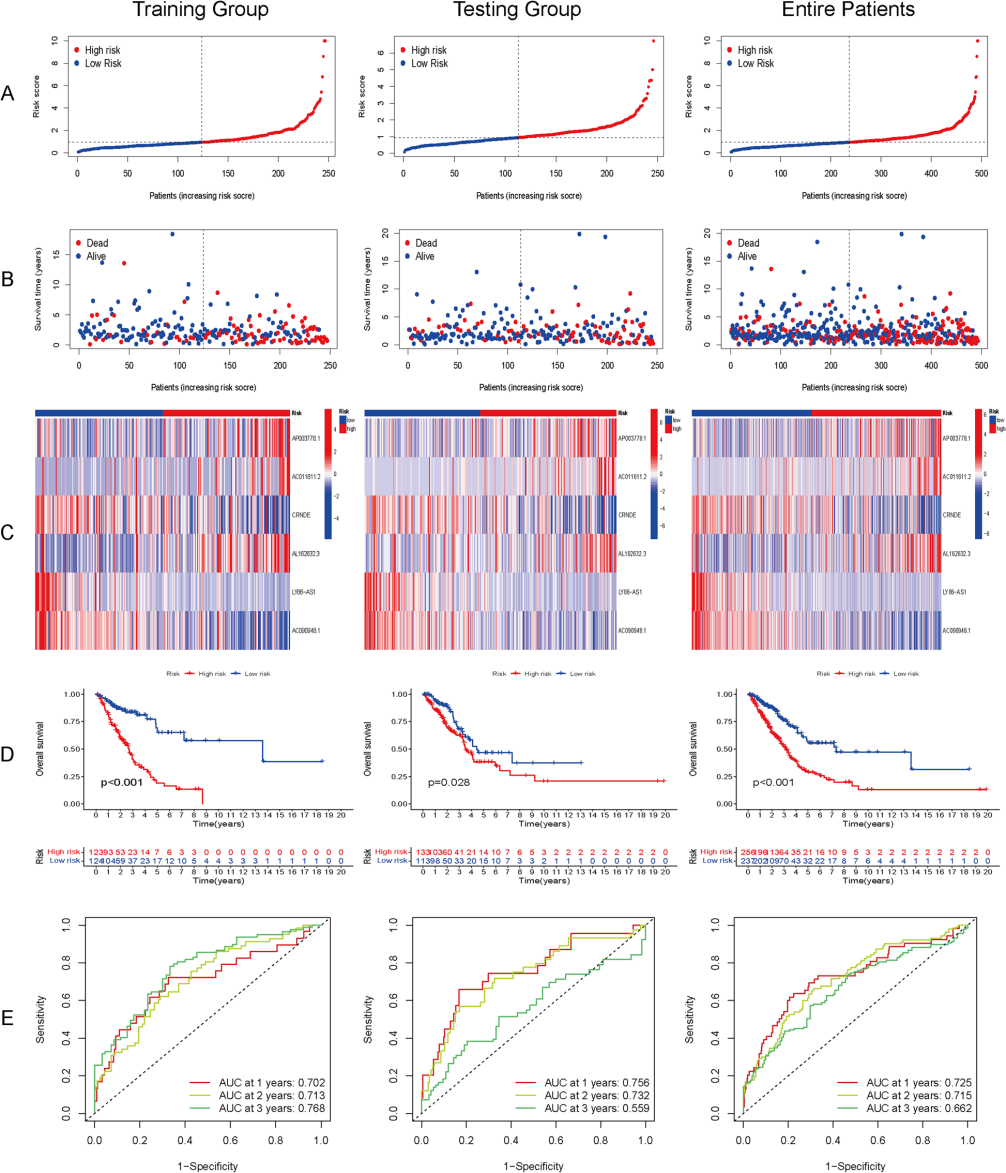
Prognostic analysis of the cuproptosis-related lncRNA signature in the training group, testing group and entire patients. **A** The distribution of the risk scores in the three groups. **B** The distributions with concomitant risk scores of overall survival status and overall survival in the three groups. **C** Correlation of the expression of the six cuproptosis-related lncRNAs and risk score in the three groups. **D** Kaplan–Meier curves for overall survival in the three groups showed that a high risk score indicated poor prognosis. **E** AUC curve analysis showed the 1-, 2-, and 3-year survival rates in the three groups

The calculation formula established in the training group analysis was used to compute the risk score for the testing group, and the same cutoff value was used to group the testing group and entire patients. The comparison of risk scores and their distributions between the testing group, entire patient cohort, and training group, as demonstrated in Fig. 3A and B, indicate no substantial discrepancies. The expression of the six lncRNAs (Fig. 3C) and KM curves for OS (Fig. 3D) in the three groups (training, testing, and entire patients) were not significantly different. AUC analysis effectively predicted the survival rates at 1, 2, and 3 years in the three groups (AUC value at 1, 2, and 3 years: training, 0.702, 0.713, 0.768; testing, 0.756, 0.732, 0.559; entire patients, 0.725, 0.715, 0.662) (Fig. 3E). The high-risk group showed similar lncRNA expression, distribution, function and poor prognosis in the three groups. These results confirmed that our signature was reliable.

### Construction of a nomogram for predicting OS

**Fig. 4:**
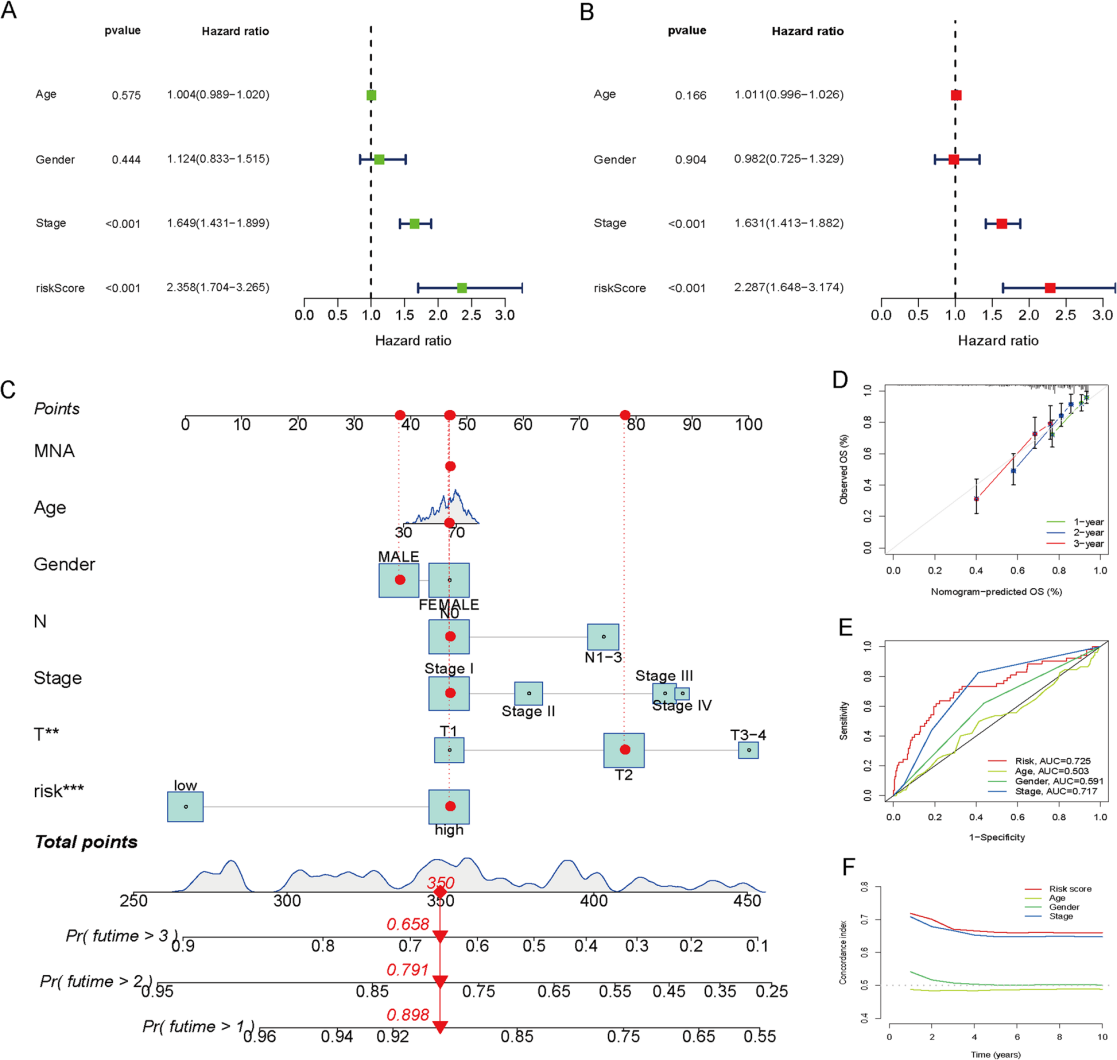
Validation of the signature containing six cuproptosis-related lncRNAs to predict prognosis. **A** Results of univariate Cox regression analysis. **B** Results of multivariate Cox regression analysis. **C** Prognostic nomogram to quantitatively predict the 1-, 2-, and 3-year survival times based on risk score and clinical characteristics. **D** Calibration curves were used to evaluate the consistency of the nomogram. **E** The results of ROC curve analysis comparing the accuracy of the risk score and clinical characteristics. **F** C-index analysis comparing the accuracy of the risk score and clinical characteristics

The results of univariate Cox analysis indicated significant correlation between risk score and prognosis in LUAD (HR = 2.358, 95% CI = 1.704–3.265, *p* ˂ 0.001) (Fig. 4A), which remained significant after adjusting confounding factors in multivariable Cox analysis (HR = 2.287, 95% CI = 1.648–3.174, *p* ˂ 0.001) (Fig. 4B). To provide clinicians with a reliable tool for predicting survival times in LUAD patients, we constructed a nomogram-based prognostic model that utilizes the risk score and clinical characteristics to provide a quantitative forecast of the 1-, 2-, and 3-year OS (Fig. 4C). Based on the calibration curves, the prognostic nomogram was found to provide reliable estimates of patient survival probabilities, with high consistency between predicted and actual outcomes (Fig. 4D). Our analysis of the ROC curves revealed that the AUC value associated with the signature was significantly higher than that of clinical predictors of prognosis for 1-year OS prediction (Fig. 4E). Index of concordance (C-index) analysis confirmed the signature's accuracy (Fig. 4F). The signature effectively distinguished between two risk groups according to principal component analysis (PCA) (Supplementary Fig. 2). The prognostic analysis confirmed the signature's capability to effectively predict both OS and progression-free survival (PFS) in patients with LUAD (Supplementary Fig. 3).

### Enrichment analysis

**Fig. 5:**
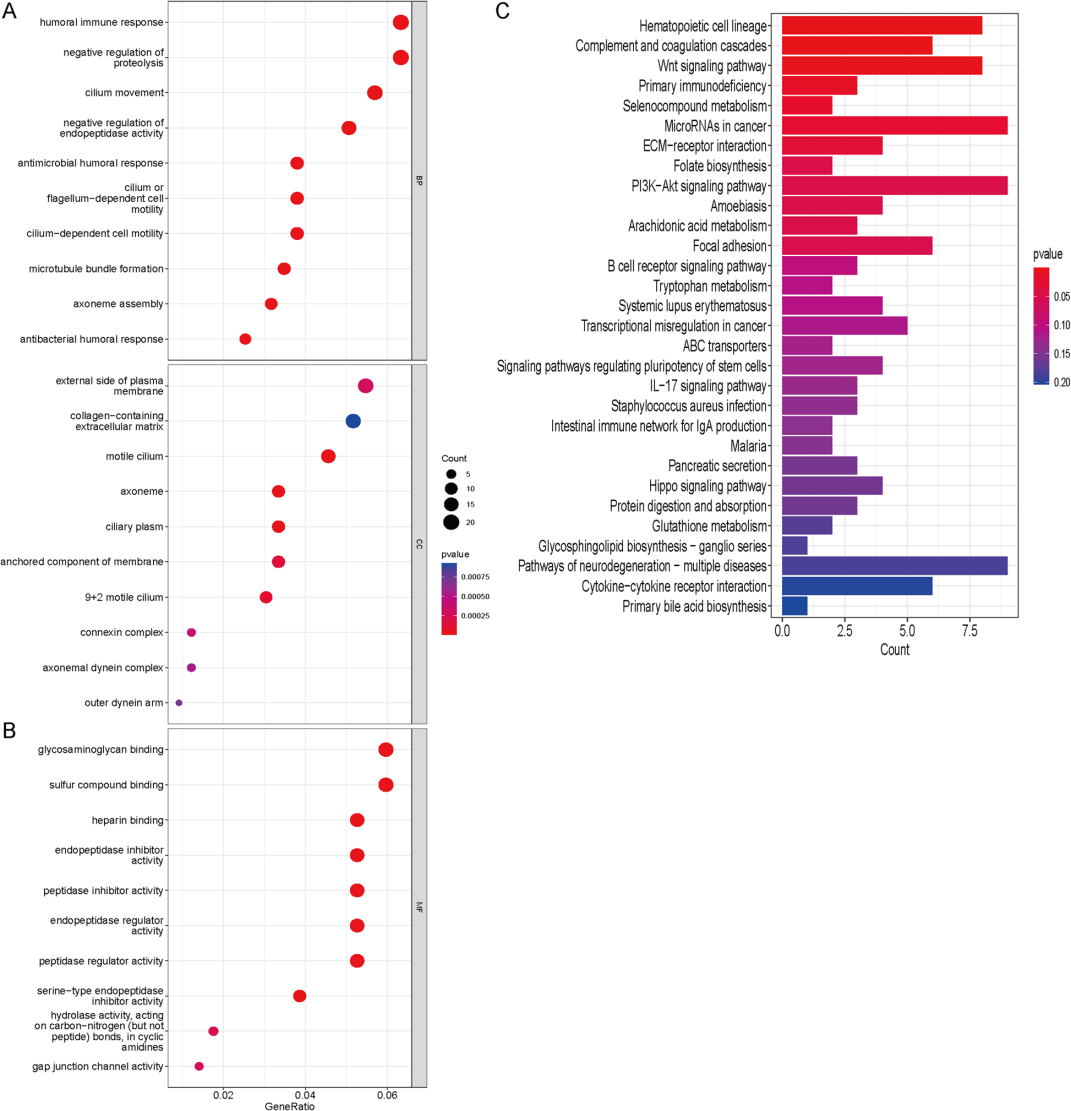
Results of GO and KEGG enrichment analyses. **A**, **B** Bubble diagram of GO analysis of differentially expressed genes. **C** Histogram of KEGG analysis of differentially expressed genes

The bubble diagram of GO analysis showed that DEGs were involved in various BPs, MFs and CCs, especially in the humoral immune response and negative regulation of proteolysis (Fig. 5A, B). The KEGG analysis histogram indicated that DEGs were implicated in a variety of signaling pathways, especially in the PI3K − Akt pathway and microRNAs in cancer (Fig. 5C).

### Relationship of risk scores with immune infiltration and response to immunotherapy

**Fig. 6:**
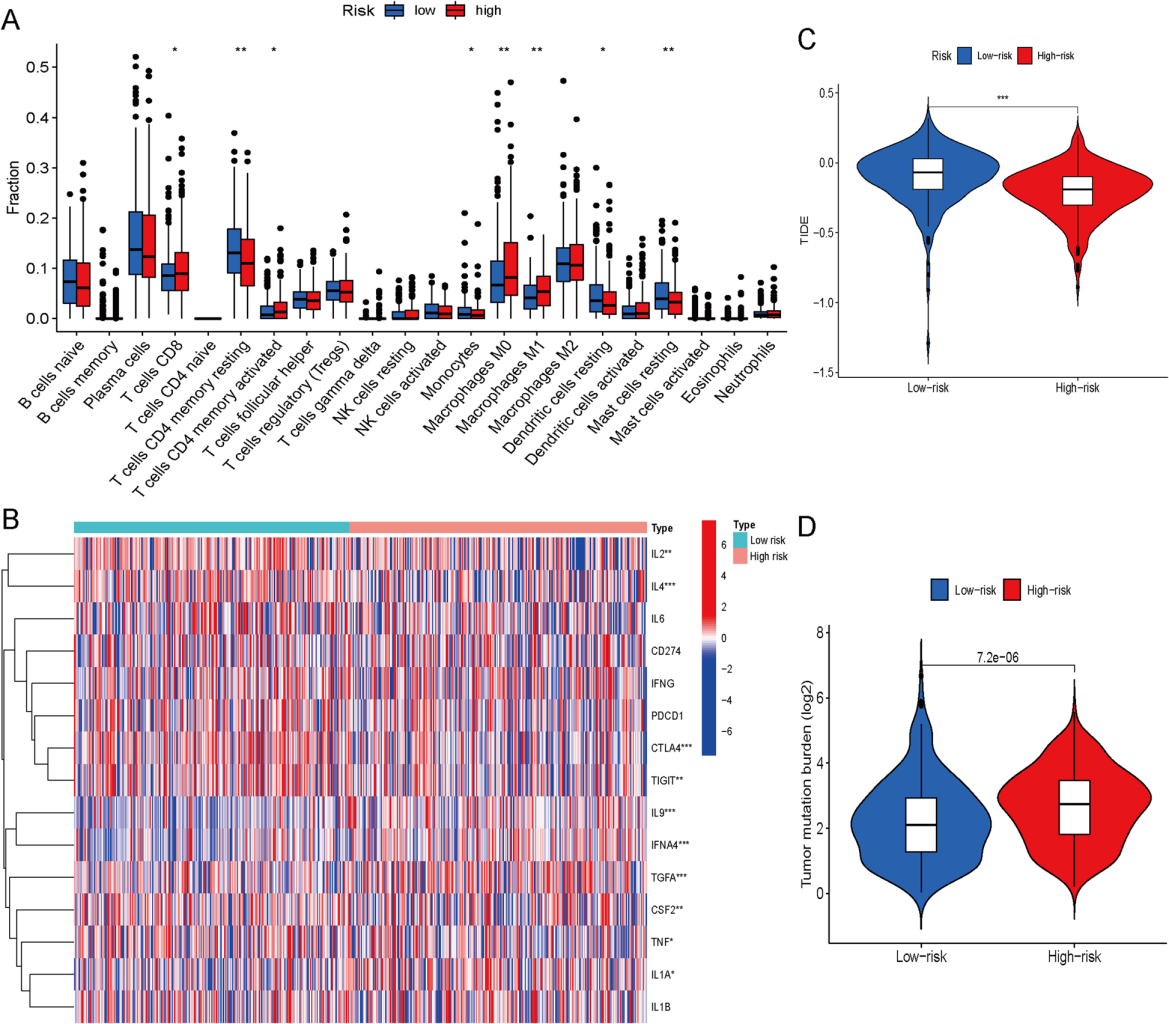
Role of risk scores in immune infiltration and immunotherapy response. **A** The relationship between the risk score and multiple infiltrating immune cells. **B** The relationship between the risk score and multiple inflammatory factors and immune checkpoints. **C** TIDE scores in the high-risk and low-risk groups. **D** Comparison of TMB levels between the high-risk group and the low-risk group. (* *p* ˂ 0.05, ** *p* ˂ 0.01, *** *p* ˂ 0.001)

The histogram revealed a correlation between the risk score and multiple immunocyte infiltration in LUAD patients (Fig. 6A). The high-risk group exhibited a notable increase in levels of CD8 + T cells and CD4 + memory T cells, which may be beneficial to immunotherapy. The heatmap revealed a correlation between the risk score and various immune checkpoints and inflammatory factors, including IL2, IL4, CTLA4 and TIGIT (Fig. 6B). Both anti-CTLA4 and anti-TIGIT inhibitors have been applied in the clinic and have shown strong antitumor ability. Inflammatory biomarker levels including IL2 and IL4 are related to the response to immunotherapy [19]. This result suggested that the risk score affects immunotherapy response. Patients with an elevated risk showed lower TIDE scores (Fig. 6C). The TIDE score is positively correlated with immune escape. This result suggested and verified that immunotherapy may be more effective for patients with higher risk scores due to lower immune escape. The higher the risk score, the higher the TMB (Fig. 6D). The high level of TMB is a marker of immunotherapy response, and confirmed again the above conclusion.

### Screening potential antitumor drugs

**Fig. 7:**
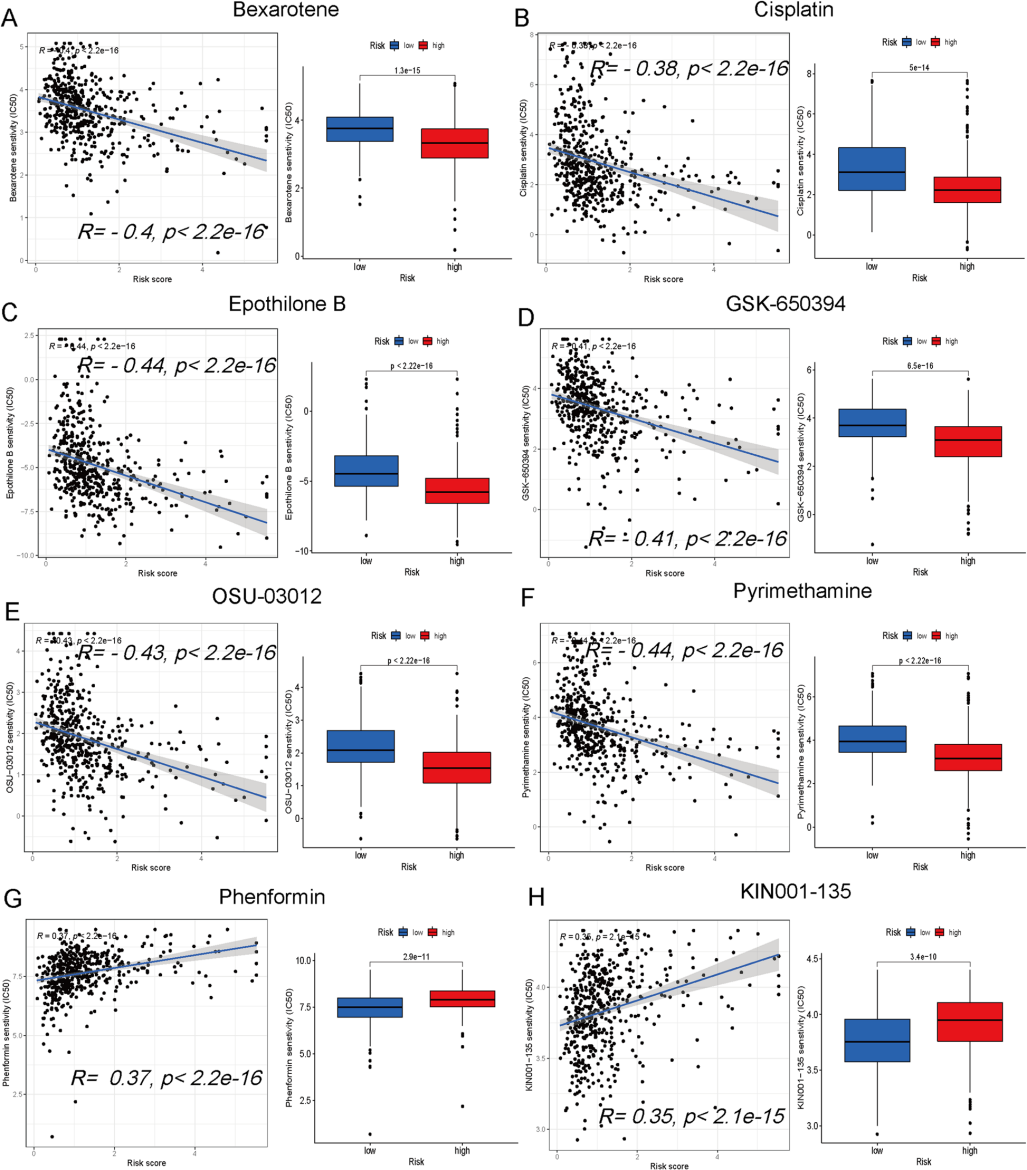
Correlation between the risk score and antitumor drug sensitivity. The correlation of the risk score with antitumor drug sensitivity, including a negative correlation with bexarotene (**A**), cisplatin (**B**), epothilone B (**C**), GSK-650394 (**D**), OSU-03012 (**E**) and pyrimethamine (F), and a positive correlation with phenformin (**G**) and KIN001-135 (**H**)

The dot plots show the correlation and *p* value between the risk score and drug IC50. A lower IC50 indicates higher drug sensitivity. Multiple drugs were correlated with risk scores, including a negative correlation with bexarotene (Fig. 7A), cisplatin (Fig. 7B), epothilone B (Fig. 7C), GSK-650394 (Fig. 7D), OSU-03012 (Fig. 7E) and pyrimethamine (Fig. 7F) and a positive correlation with phenformin (Fig. 7G) and KIN001-135 (Fig. 7H). Additional drug associations with the risk score are presented in Supplementary Fig. 4.

## Discussion

This study comprehensively evaluated the significance of cuproptosis-related lncRNAs in predicting the immunotherapy response and prognosis of LUAD patients, and a prognostic risk signature with six cuproptosis-related lncRNAs was developed. In predicting the prognosis of LUAD patients, the signature outperformed clinical characteristics in accuracy. The enrichment analysis of DEGs showed that there were different biological functions and signaling pathways in two risk groups. The immune infiltration patterns varied between the high-risk and low-risk groups, with the former showing potential for immunotherapy due to their lower rate of immune evasion and higher level of TMB. In addition, accurately predicting the efficacy of multiple potential antitumor drugs is possible using the risk score.

We studied 19 genes associated with cuproptosis from Tsvetkov et al.’s research [7]. The full names of 19 genes are listed in Supplementary Table 1. Specifically, NFE2L2 is involved in mismatch repair (MMR) and DNA methyltransferase expression in human pancancer [20]. Excessive copper regulates the inflammatory response via NLRP3 expression [21]. MTF1 promoted ATP7B expression [22]. ATP7A and ATP7B are two homologous copper transporter genes that are involved in maintaining cellular copper homeostasis [23]. SLC31A1 is a copper transporter that promotes copper absorption [24]. FDX1 is involved in cuproptosis [25], and FDX1 knockout results in the loss of DLAT and DLST expression [7]. Mitochondrial lipoate synthesis involves three enzymes, LIAS, LIPT2 and LIPT1 [26]. Pyruvate dehydrogenase complex (PDHc) defects are mitochondrial disorders involving six related genes (PDHA1, PDHB, DLAT, DLD, PDHX, and PDP1) [27]. GLS promotes copper excretion [28]. CDKN2A expression correlates with peripheral blood copper levels in people occupationally exposed to copper [29]. Copper promotes tumor proliferation, angiogenesis, and metastasis [9]. These results showed that cuproptosis-related genes regulated copper levels to affect tumor prognosis through multiple mechanisms.

LncRNAs regulate gene transcription and protein expression but are not involved in coding proteins. Most cancers are linked to the malfunction of programmed cell death, and recent research has demonstrated that lncRNAs are essential players in modulating this process [30]. Studies indicate that lncRNA AFAP1-AS1 upregulation was linked to an increase in anti-apoptotic protein Bcl-2 expression and a decrease in apoptosis in NSCLC [31]. A pyroptosis-related lncRNA signature has been shown to be correlated with LUAD prognosis and immune infiltration [32]. Via EGFR/MET/STAT3 signal transduction, lncRNA LOC389641 inhibits autophagy by decreasing the levels of the autophagic markers p-AMPK and LC3B, which contributes to its oncogenic activity in LUAD [33]. Ferroptosis-related lncRNAs affect antitumor immunity [16]. Aberrant lncRNA levels may be important cancer-specific diagnostic markers [34]. Moreover, in vivo experimental studies recently showed that by modulating PICALM splicing through SRSF6, lncRNA CRNDE enhances the response of gastric cancer cells to chemotherapy [35]. LUAD patients with increased expression of LY86-AS1 tend to have a positive prognosis [36]. AC090948.1 is involved in the immune infiltration of bladder cancer [37]. Consistent with previous studies, our study identified six cuproptosis-related lncRNAs (including AP003778.1, AC011611.2, CRNDE, AL162632.3, LY86-AS1, and AC090948.1) that were demonstrated a significant link with clinical outcomes of LUAD patients (Supplementary Fig. 1). In the entire database and in the training/testing group, AUC and ROC analysis well predicted the survival rates than clinical predictors of prognosis. Furthermore, the calibration curve demonstrated excellent agreement between predicted and observed outcomes.

Cuproptosis characterizes a newly recognized mitochondrial cell death caused by intracellular copper accumulation [38, 39]. Copper's promotion of angiogenesis is achieved through a combination of increased migration and proliferation of endothelial cells, and enhanced synthesis of fibronectin [40]. Angiogenesis promotes tumor proliferation and metastasis, resulting in poor prognosis. Several traditional copper chelators (such as penicillamine and captopril) have been used to inhibit angiogenesis by reducing the copper concentration [41]. The standard therapy for NSCLC includes antiangiogenic therapy, which have been proven to significantly extend patient survival. Copper complexes (such as tetrathiomolybdate and chloroquinoline) have been reutilized for cancer treatment due to anticancer activity in cellular and animal models [42]. A decrease in serum copper levels increases IL1β, IL4, IL6, IL18 and IFN-γ levels [10]. Studies have shown that intratumoral copper levels promote PD-L1 expression and tumor immune escape [43]. In our study, enrichment analysis revealed that DEGs were concentrated in the humoral immune response, negative regulation of proteolysis, microtubule − based movement, PI3K − Akt pathway and the microRNAs in cancer. PI3K − Akt signaling pathway activation promotes tumor proliferation and inhibits PD-L1 expression [44]. The humoral immune response is connected to antitumor immunity and the immunotherapy response. Therefore, our signature may be related to the immunotherapy response.

Immunotherapy has brought new hope to malignant tumor patients [45]. Immune escape is one of the top ten challenges of cancer immunotherapy [46]. Our findings indicated that the risk score was related to multiple inflammatory factors and immune checkpoints, including IL2, IL4, CTLA4 and TIGIT, and patients with a higher score may exhibit lower immune escape. Immunotherapy may be selected in advance for LUAD patients with lower immune escape based on the risk score. A higher TMB level is a marker for predicting the efficacy of immunotherapy [47]. Higher risk patients had significantly higher TMB in our study, suggesting a potential advantage for immunotherapy. The risk score also predicted drug sensitivity. Chemotherapy and targeted treatment represent the primary treatment methods for LUAD. According to our research, individuals displaying elevated risk scores exhibit greater sensitivity towards bexarotene, cisplatin, epothilone B and pyrimethamine. Chemotherapy- and targeted therapy-related side effects are closely related to the dose [48]. The clinical chemotherapy and targeted drug dose may be adjusted according to the drug sensitivity of LUAD patients assessed by risk scores to reduce side effects. In conclusion, our risk score signature can be used to predict immune infiltration, immune escape, drug sensitivity and prognosis, providing guidance for the clinical management of LUAD patients.

Our research has some limitations. Confirming the prognostic risk signature presented in this study requires the use of real-world, multicenter, and prospective data. The specific mechanism by which cuproptosis-related lncRNAs contribute to immune infiltration requires conducting biological experiments on both cellular and animal models.

## Conclusion

Based on cuproptosis-related lncRNAs, we constructed and validated a novel risk signature that may be used to predict immunotherapy efficacy and prognosis in LUAD patients.

## Supplementary Information

**Additional file 1:** **Supplementary Figure 1.** The six cuproptosis-related lncRNAs were significantly correlated with the prognosis of LUAD patients.

**Additional file 2:** **Supplementary Figure 2.** The results of principal component analysis.

**Additional file 3:** **Supplementary Figure 3.** (A, B) The risk score well predicted the prognosis of early-stage or advanced-stage LUAD patients. (C) The risk score well predicted the PFS of LUAD patients.

**Additional file 4:** **Supplementary Figure 4.** The correlation between various drugs and the risk score.

**Additional file 5:** **Supplementary Table 1.** Lifestyle-related questionnaire.

## Data Availability

The data of this study are from the public TCGA database and can be obtained at this link (https://portal.gdc.cancer.gov/repository).

## Abbreviations

LncRNAs: Long noncoding RNAs
TCGA: The Cancer Genome Atlas
LUAD: Lung adenocarcinoma
NSCLC: Non-small cell lung cancer
DEGs: Differentially expressed genes
GO: Gene Ontology
KEGG: Kyoto Encyclopedia of Genes and Genomes
TMB: Tumor mutational burden
TIDE: Tumor Immune Dysfunction and Exclusion
PD-1/PD-L1: Programmed cell death protein-1 and its ligand
