# Supplementary figures and images for: A cuproptosis-related lncRNA signature for predicting prognosis and immunotherapy response of lung adenocarcinoma
Source: Hereditas. 2023 Jul 24;160:31. doi: 10.1186/s41065-023-00293-w (PMC10364405; doi:10.1186/s41065-023-00293-w)

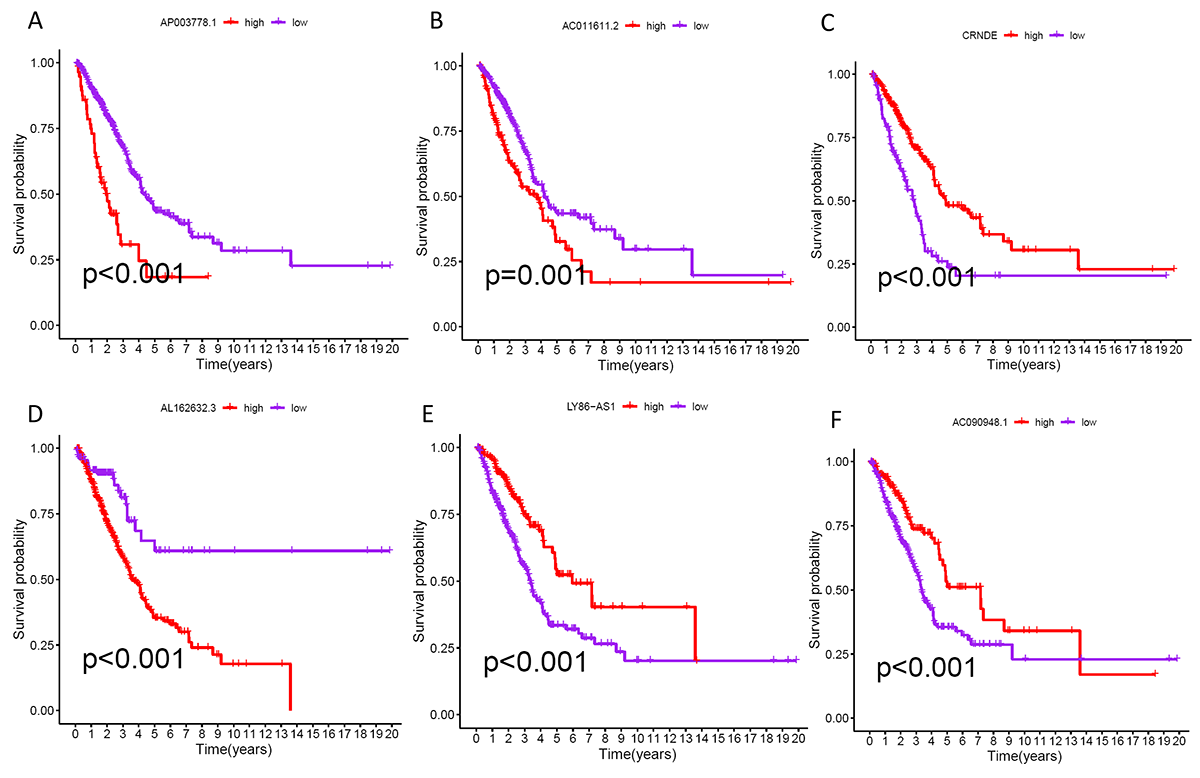

Supplement: Supplementary file 1 — Additional file 1: Supplementary Figure 1. The six cuproptosis-related lncRNAs were significantly correlated with the prognosis of LUAD patients. [file 41065_2023_293_MOESM1_ESM.tif]

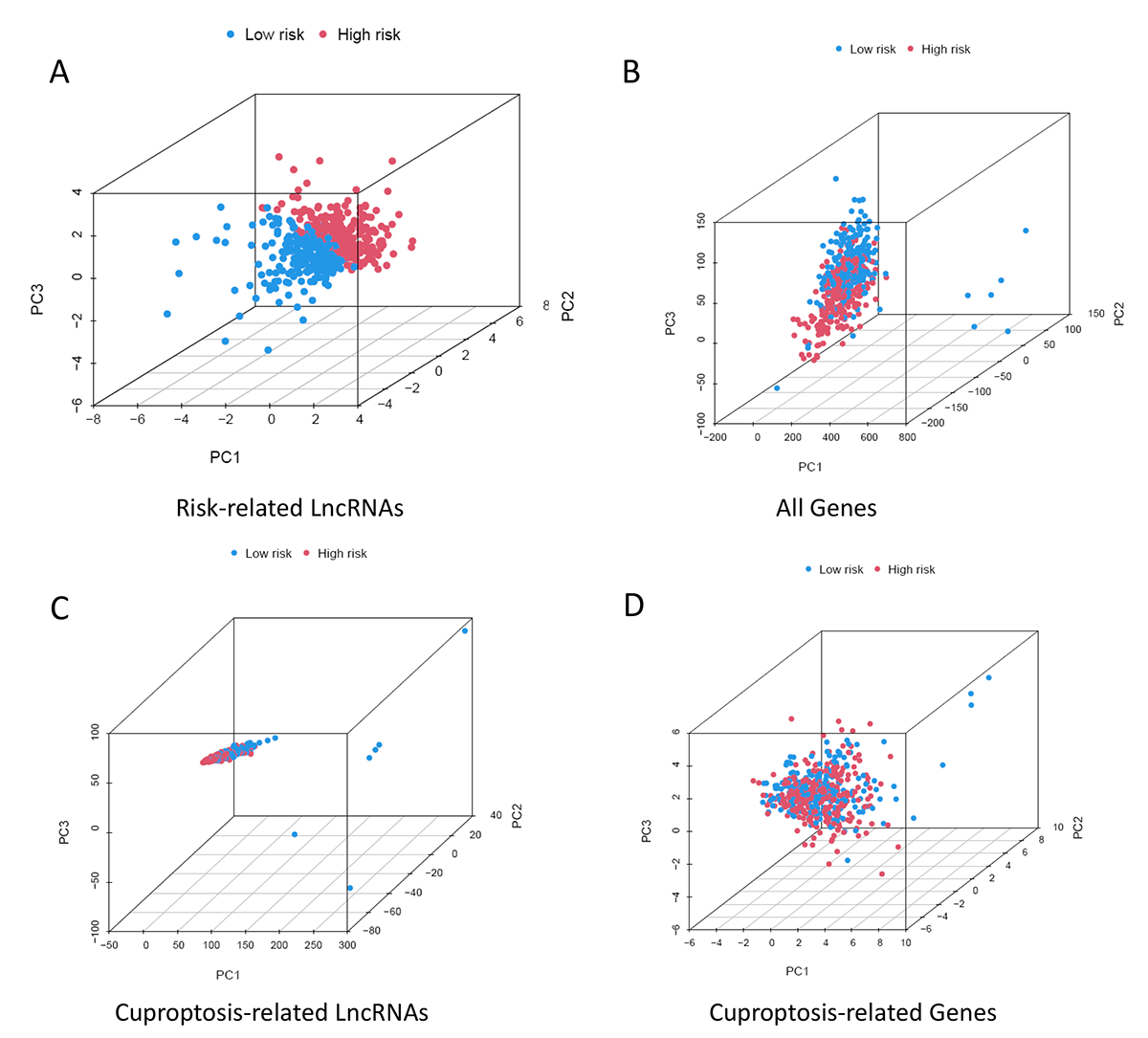

Supplement: Supplementary file 2 — Additional file 2: Supplementary Figure 2. The results of principal component analysis. [file 41065_2023_293_MOESM2_ESM.tif]

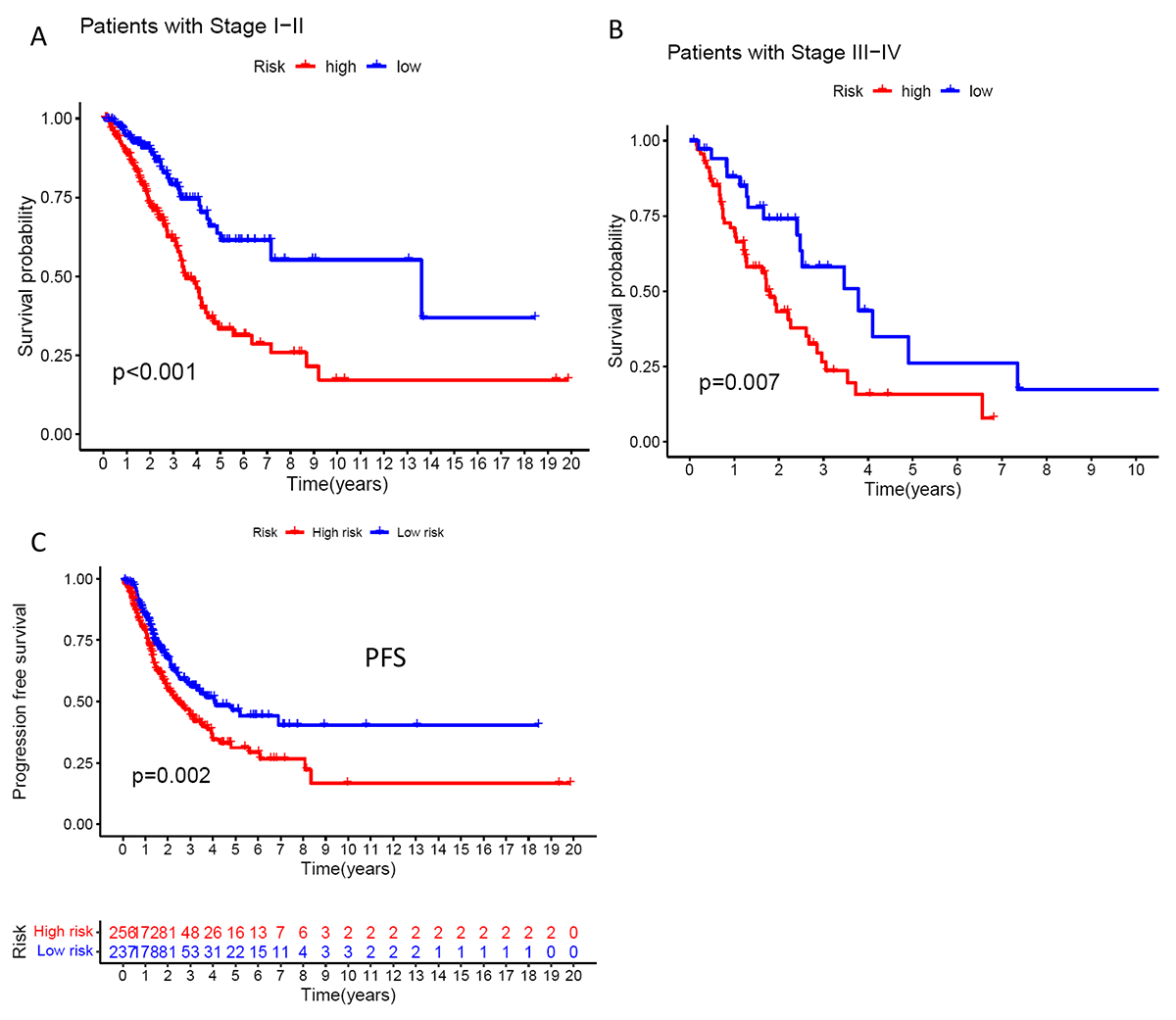

Supplement: Supplementary file 3 — Additional file 3: Supplementary Figure 3. (A, B) The risk score well predicted the prognosis of early-stage or advanced-stage LUAD patients. (C) The risk score well predicted the PFS of LUAD patients. [file 41065_2023_293_MOESM3_ESM.tif]

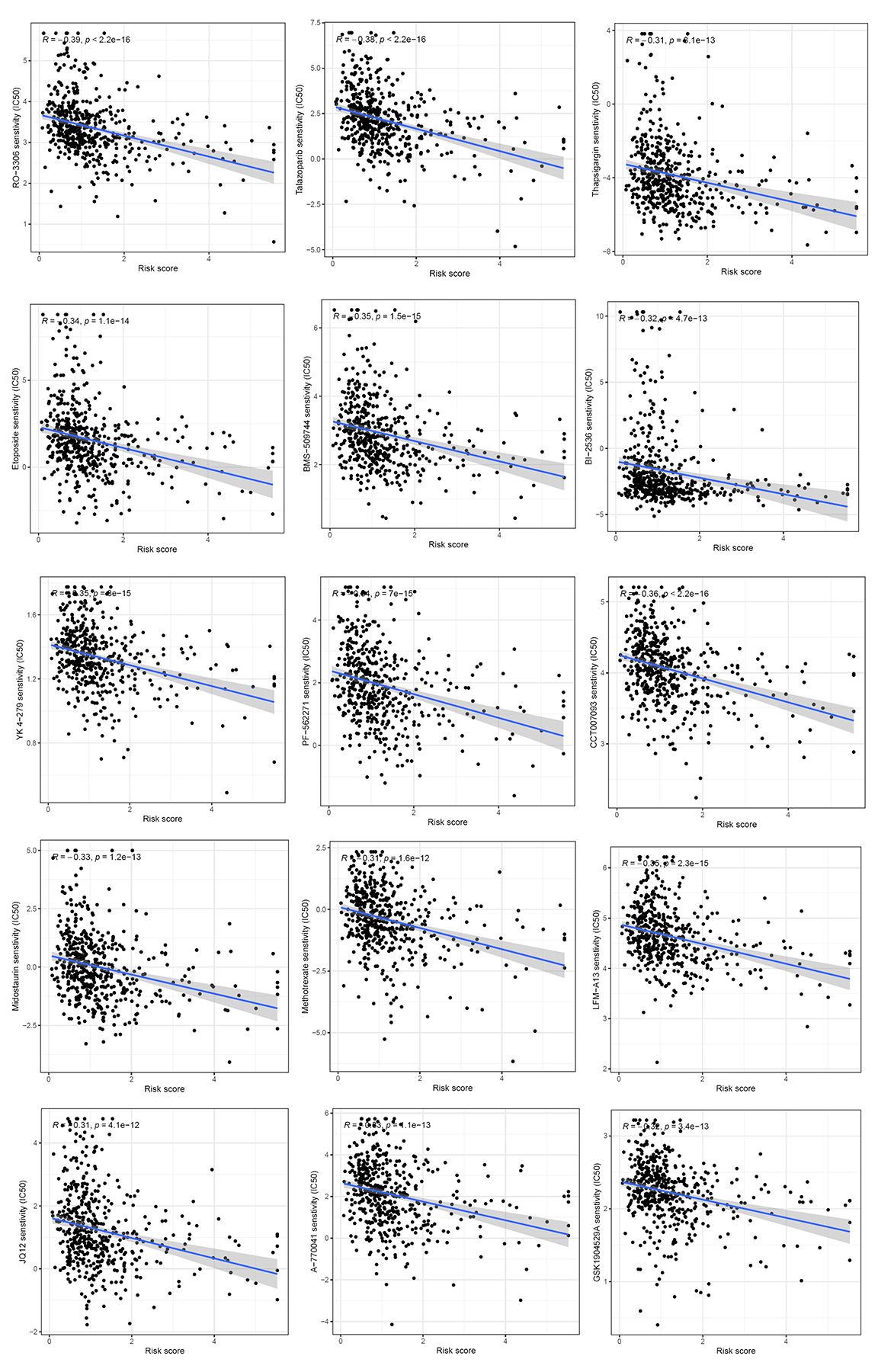

Supplement: Supplementary file 4 — Additional file 4: Supplementary Figure 4. The correlation between various drugs and the risk score. [file 41065_2023_293_MOESM4_ESM.tif]
