# Supplementary material for: A cuproptosis-related lncRNA signature for predicting prognosis and immunotherapy response of lung adenocarcinoma
Source: Hereditas. 2023 Jul 24;160:31. doi: 10.1186/s41065-023-00293-w (PMC10364405; doi:10.1186/s41065-023-00293-w)
Supplement: Supplementary file 5 — Additional file 5: Supplementary Table 1. Lifestyle-related questionnaire. [file 41065_2023_293_MOESM5_ESM.docx]

**Supplementary materials**

Supplementary Table 1. Cuproptosis-related genes

| Gens | Full name |
| --- | --- |
| NFE2L2 | nuclear factor erythroid 2-related factor 2 |
| NLRP3 | Nod-like receptor pyrin domain containing 3 |
| ATP7B | adenosine triphosphatase copper transporting beta |
| ATP7A | adenosine triphosphatase copper transporting alpha |
| SLC31A1 | solute carrier family 31 member 1 |
| DLST | dihydrolipoamide S-succinyltransferase |
| LIAS | lipoic acid synthase |
| FDX1 | ferredoxin 1 |
| LIPT1 | lipoyl(octanoyl) transferase 1 |
| LIPT2 | lipoyl(octanoyl) transferase 2 |
| DLD | dihydrolipoamide dehydrogenase |
| DLAT | dihydrolipoyllysine acetyltransferase |
| PDHA1 | pyruvate dehydrogenase alpha 1 |
| PDHB | pyruvate dehydrogenase beta |
| MTF1 | metal-regulatory transcription factor-1 |
| GLS | glutaminase |
| CDKN2A | cyclin-dependent kinase inhibitor alpha |
| DBT | dihydrolipoamide branched chain transacylase |
| GCSH | glycine cleavage system protein H |
